# Short-term and long-term outcomes after robotic radical surgery for rectal gastrointestinal stromal tumor

**DOI:** 10.1186/s12893-024-02434-y

**Published:** 2024-05-09

**Authors:** Chikara Maeda, Yusuke Yamaoka, Akio Shiomi, Hiroyasu Kagawa, Hitoshi Hino, Shoichi Manabe, Chen Kai, Kenji Nanishi

**Affiliations:** Division of Colon and Rectal Surgery, Shizuoka Cancer Center Hospital, 1007 Shimonagakubo, Nagaizumi-Cho, Sunto-Gun, Shizuoka, 411-8777 Japan

**Keywords:** Gastrointestinal stromal tumor (GIST), rectal GIST, Robotic surgery, Sphincter preservation

## Abstract

**Background:**

The optimal approach for ensuring both complete resection and preservation of anal function in rectal gastrointestinal stromal tumor (GIST) remains unknown. The aim of this study was to clarify short-term and long-term outcomes after robotic radical surgery for rectal GIST.

**Methods:**

A total of 13 patients who underwent robotic radical surgery for rectal GIST between December 2011 and April 2022 were included. All robotic procedures were performed using a systematic approach. A supplemental video of robotic radical surgery for rectal GIST is attached. The short-term outcome was the incidence of postoperative complications during the first 30 days after surgery. Surgical outcomes were retrieved from a prospective database. Long-term outcomes, including overall survival and recurrence-free survival, were determined in all patients.

**Results:**

Median distance from the tumor to the anal verge was 4.0 cm. Surgical margins were negative in all patients. Two patients underwent neoadjuvant imatinib therapy. All patients underwent sphincter-preserving surgery. None underwent conversion to open or laparoscopic surgery. The incidence of postoperative Clavien-Dindo grade II and grade ≥ III complications was 7.7% and 0%, respectively. The median postoperative hospital stay was 7 days. Twelve patients (92.3%) underwent stoma closure within 5 months of the initial surgery. Median follow-up time was 76 months. The 5-year overall survival and recurrence-free survival rates were both 100%. None of the patients had recurrence.

**Conclusion:**

Short-term and long-term outcomes after radical robotic surgery for rectal GIST were favorable. Robotic surgery might be a useful surgical approach for rectal GIST.

**Supplementary Information:**

The online version contains supplementary material available at 10.1186/s12893-024-02434-y.

## Introduction

Gastrointestinal stromal tumors (GISTs) are the most common mesenchymal neoplasms of the gastrointestinal tract [1, 2]. However, rectal GIST is extremely rare, making up approximately 0.1% of all rectal neoplasms and approximately 5% of all GISTs [3, 4]. The standard treatment for localized GIST is complete surgical excision [5]. For rectal GIST, it has been debated whether radical surgery or local resection is appropriate as surgical treatment [6]. A recent study enrolling more than 200 patients with rectal GIST reported that radical resection is superior to local resection for rectal GIST of > 2 cm in terms of oncological outcomes [7]. Regarding radical resection for rectal GIST, whether open, laparoscopic, robotic, or transanal surgery is the optimal surgical approach has not been discussed. To date, only a few case reports have been published about each approach [8–11]. Therefore, the optimal approach for ensuring both complete resection and preservation of anal function remains unknown. This study was conducted to clarify the short-term and long-term outcomes after robotic radical surgery for rectal GIST.

## Material and methods

### Patient selection

Patients who underwent robotic radical surgery for rectal GIST at Shizuoka Cancer Center in Japan between December 2011 and April 2022 were included. The institutional review board of Shizuoka Cancer Center Hospital approved data collection and analysis (institutional code: J2022-20). Patient characteristics, including age, sex, body mass index, tumor size, tumor distance from the anal verge, presence of distant metastasis, and presence of adjuvant or neoadjuvant imatinib therapy were recorded in a prospective database. Preoperative evaluation consisted of colonoscopy, endorectal ultrasonography, computed tomography (CT), and magnetic resonance imaging. Fine needle aspiration or needle biopsy was performed for diagnosis. Recurrence risk was assessed using the modified Fletcher classification [12].

### Treatment

In accordance with the Japanese Clinical Practice Guidelines for GISTs [13], primary resectable GIST was typically treated with complete resection without injury to the pseudocapsule and macroscopically negative margins. When complete resection was considered difficult to achieve with transanal local resection, transabdominal radical resection was selected. Robotic surgery was introduced in December 2011. The indication for robotic surgery was any rectal GIST. Robotic surgery for rectal malignant tumors was not covered by national health insurance in Japan until March 2018. Therefore, it was a costlier treatment option than laparoscopic or open surgery. After providing informed consent, robotic, laparoscopic, or open surgery was selected on the basis of patient preference. Preoperative imatinib therapy was performed only in patients for whom complete resection without imatinib therapy was predicted to be difficult or when shrinkage of the tumor with imatinib therapy would make anal preservation possible or would permit the avoidance of urinary diversion. Intersphincteric resection was performed when the rectum could not be divided using linear staplers via the abdominal approach. If the tumor invaded the levator ani muscle or fecal continence was impaired, abdominoperineal resection was performed. Adjuvant imatinib therapy was performed for 3 years in patients categorized as being in the high-risk group based on the modified Fletcher classification. All treatment strategies, including operative approaches or procedures, were approved at a multidisciplinary team conference at our institution.

### Operative technique

**Fig. 1:**
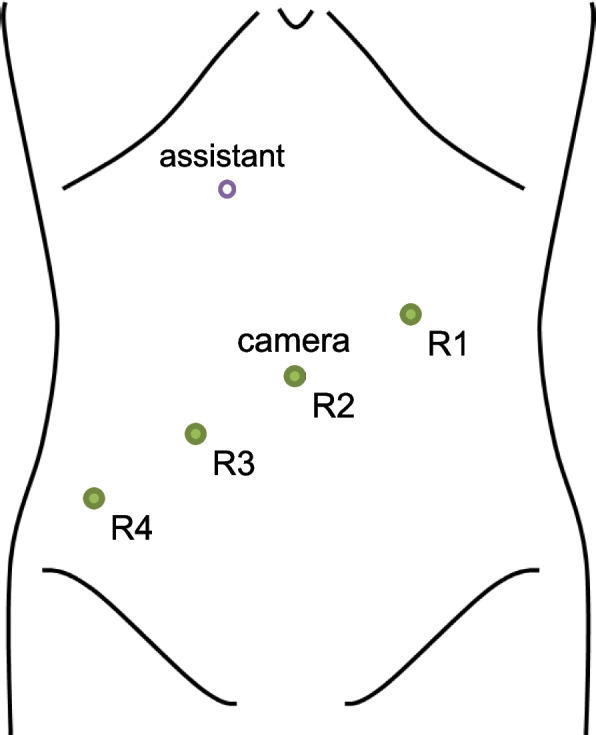
Trocar placement. R1, R2, R3, and R4 refer to robotic arms 1, 2, 3, and 4, respectively

All robotic procedures were performed using a systematic approach. Trocars were placed as shown in Fig. 1. The rectum was mobilized down to the pelvic floor with sharp dissection in front of the fascia of the prehypogastric nerve and behind Denonvilliers’ fascia to avoid autonomic nerve injury if oncologically safe [14, 15]. If tumor invasion beyond the mesorectum was suspected, en bloc resection of adjacent organs or pelvic autonomic nerves was performed. The inferior mesenteric artery was preserved and central lymph node dissection was not performed because lymph node metastasis is rarely seen in GIST [12]. The supplemental materials include a video of robotic radical surgery for rectal GIST.

### Surveillance protocol

Surveillance was performed for 10 years after surgery. The surveillance protocol at our institution consisted of an interview, physical examination, blood tests, and CT of the chest, abdomen, and pelvis every 6 months. Colonoscopy was performed annually for the first 3 years after surgery. Recurrence was confirmed pathologically or based on progressively increasing tumor size in imaging studies.

### Outcome measurements

The short-term outcome was the incidence of postoperative complications during the first 30 days after surgery, based on the Clavien-Dindo classification [16]. Urinary dysfunction was defined as residual urine volume of more than 50 mL. The following surgical outcomes were retrieved from the prospective database: intraoperative blood loss, operative time, conversion to open surgery, days to soft diet, and postoperative hospital stay. The rate of complete resection was also gathered. Long-term outcomes, including overall survival (OS) and recurrence-free survival (RFS), were determined in all patients.

### Statistical analysis

Categorical variables are presented as numbers (percentages). Continuous variables are presented as medians (range). OS and RFS rates were calculated using the Kaplan–Meier method. All statistical analyses were performed using EZR software, version 1.40 (Saitama Medical Center, Jichi Medical University, Saitama, Japan) [17], which is a graphical user interface for R (The R Foundation for Statistical Computing, Vienna, Australia).

## Results

### Patient characteristics

During the study period, 13 patients underwent robotic radical surgery for rectal GIST. Their clinicopathological characteristics are presented in Table 1. There were nine males and four females. The median age was 58 (range, 39–72) years. The median distance from the tumor to the anal verge was 4.0 (range, 1.7–5.0) cm. Two patients underwent neoadjuvant imatinib therapy. Surgical margins were negative in all patients. All 13 GISTs stained positive for CD117 (c-kit) and CD34. According to the modified Fletcher classification [12], nine patients (69.2%) were in the very-low-risk or low-risk group, one patient (7.7%) was in the intermediate risk group, and three patients (23.1%) were in the high-risk group.

**Table 1:** Clinicopathological characteristics of the study patients (*n* = 13)

Characteristic	*n* = 13
Age, year [median (range)]	58 (39–72)
Sex	
Male	9 (69.2)
Female	4 (30.8)
Body mass index, kg/m^2^ [median (range)]	23.0 (19.1–28.4)
Tumor distance from anal verge, cm [median (range)]	4.0 (1.7–5.0)
Distant metastasis	0 (0)
Neoadjuvant imatinib therapy	2 (15.4)
Adjuvant imatinib therapy	3 (23.1)
Surgical margin	
R0	13 (100)
R1	0 (0)
R2	0 (0)
Tumor size, cm [median (range)]	3 (1.5–7.5)
≤ 2	1 (7.7)
2 < , < 5	10 (76.9)
≥ 5	2 (15.4)
Mitotic index, / 50HPFs	
≤ 5	11 (84.6)
5 < , < 10	1 (7.7)
≥ 10	1 (7.7)
Risk classification	
Modified Fletcher criteria	
Very low	1 (7.7)
Low	8 (61.5)
Intermediate	1 (7.7)
High	3 (23.1)

Values in parentheses represent percentages unless otherwise noted

*HPF* High-power field

### Surgical outcomes

Surgical outcomes are presented in Table 2. All patients underwent anal sphincter-preserving surgery. None underwent conversion to open or laparoscopic surgery. Five patients underwent resection of an adjacent organ. The median operative time was 288 (range, 178–306) minutes. The median blood loss was 35 (range, 0–215) mL. The incidence of postoperative Clavien-Dindo grade II and grade III or higher complications was 7.7% and 0%, respectively. None of the patients had anastomotic leakage. Among 13 patients, 12 patients (92.3%) underwent stoma closure within 5 months of initial surgery and the remaining patient did not want stoma closure.

**Table 2:** Perioperative and postoperative outcomes of the study patients (*n* = 13)

Characteristics	*n* = 13
Type of procedures	
Intersphincteric resection	11 (84.6)
Low anterior resection	2 (15.4)
Resection of the adjacent organ	5 (38.4)
Vagina	2 (15.4)
Levator ani muscle	2 (15.4)
Prostate	1 (7.7)
Diverting ileostomy	13 (100)
Conversion to open or laparoscopic surgery	0 (0)
Anastomosis level from anal verge, cm [median (range)]	2.0 (1.0–4.0)
Operative time, min [median (range)]	288 (178–306)
Blood loss, ml [median (range)]	35 (0–215)
Transfusion	0 (0)
Postoperative complications	
Anastomotic leakage	0 (0)
Intra-abdominal or intraluminal bleeding	0 (0)
Bowel obstruction	0 (0)
Urinary dysfunction	1 (7.7)
Urinary tract infection	0 (0)
Wound infection	0 (0)
Clavien-Dindo grade II	1 (7.7)
Clavien-Dindo grade III or more	0 (0)
Day to soft diet, days [median (range)]	3 (3–3)
Postoperative hospital stay, days [median (range)]	7 (6–11)
Stoma closure	12 (92.3)

Values in parentheses represent percentages unless otherwise noted

### Long-term outcomes

**Fig. 2:**
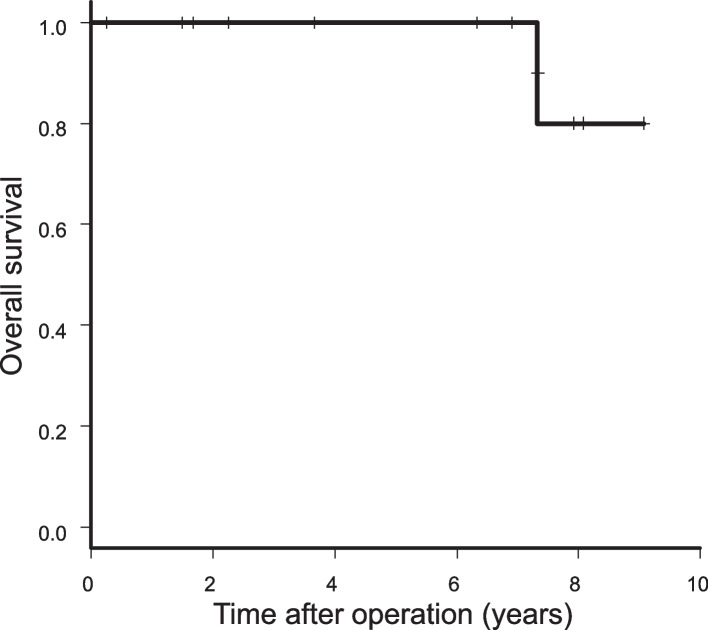
Overall survival (*n* = 13)

**Fig. 3:**
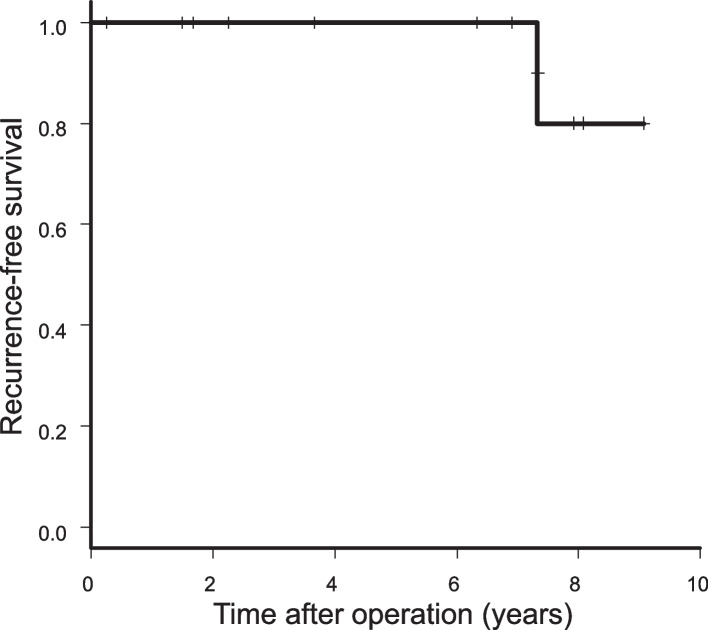
Recurrence-free survival (*n* = 13)

The median follow-up time was 76 (range, 3–109) months. Figures 2 and 3 show OS and RFS curves, respectively. The 5-year overall OS and RFS rates were each 100%. None of the patients had recurrence. One patient died of lung cancer 88 months after surgery.

## Discussion

This is the largest study evaluating the outcomes after radical surgery for rectal GIST using the robotic approach. [11]. Short-term and long-term outcomes following robotic surgery were favorable. All patients underwent sphincter-preserving surgery; the incidence of postoperative complications of Clavien-Dindo grade II and grade III or higher was 7.7% and 0%, respectively. All patients underwent R0 resection and none had recurrence. There was only one prior study reporting three patients undergoing robotic surgery for rectal GIST; their outcomes were as good as ours [11]. In radical resection for rectal GIST, two recent relatively large studies reported that the proportion of patients undergoing sphincter-preserving surgery ranged from 33 to 49%, the incidence of postoperative complications ranged from 7.0% to 22%, the rate of R0 resection ranged from 62 to 97%, and approximately 20% of patients had recurrence. The background characteristics of patients in those studies were different from those in our study and the surgical approach for radical surgery was not mentioned [6, 7]. Based on these results, robotic radical surgery for rectal GIST might be a suitable approach in terms of postoperative complications, sphincter preservation, and R0 resection.

Another minimally invasive approach is laparoscopic surgery. There were three studies reporting outcomes after laparoscopic surgery for rectal GIST; the outcomes were as favorable as ours [8–10]. However, the largest study enrolled only five patients undergoing laparoscopic surgery [9]. Therefore, it is difficult to compare robotic and laparoscopic surgery for rectal GIST. Three large randomized controlled trials have compared robotic and laparoscopic surgery for rectal cancer: ROLLAR, COLRAR, and REAL [18–20]. Although the ROLLAR and COLRAR trials did not demonstrate that robotic surgery was superior to laparoscopic surgery for their respective primary endpoints of open surgery conversion rate and total mesorectal excision quality, the REAL trial showed that the rate of circumferential resection margin positivity and the incidence of postoperative complications, which were secondary endpoints, were significantly lower with robotic surgery than with laparoscopic surgery. Multiple retrospective studies have reported that robotic surgery is superior to laparoscopic or open surgery in terms of surgical, functional, and oncological outcomes [21–26]. Surgical techniques required for radical surgery for rectal GIST are similar to those for rectal cancer surgery. However, the extent of mesenteric mobilization and the level of vascular ligation are different due to prophylactic or systematic lymph node dissection being not necessary in radical surgery for rectal GIST [27, 28]. Since rectal GIST is typically large and protruding, dissection around the tumor while maintaining a clear margin and mobilization of rectum distal to the tumor to preserve sphincter are essential. Robotic surgery via a transabdominal approach seemed to be suitable due to the following advantages: use of free-moving, multi-joint forceps; motion-scaling function; less tremor; and a stable camera with high-quality three-dimensional imaging. These factors can overcome the inherent limitations of conventional laparoscopic rectal surgery such as the use of straight, rigid instruments; limited degrees of freedom; an unstable camera with two-dimensional imaging; and poor ergonomics due to the narrow pelvic cavity and high anatomical complexity [29]. A previous study using magnetic resonance imaging-based pelvimetry reported that long sacral length, shallow sacral angle, narrow intertuberous distance, and large tumor size were significantly associated with longer pelvic dissection time in laparoscopic surgery for rectal cancer [30]. Patients were classified into three groups based on the number of these anatomical factors: easy, moderate, and difficult groups, and in patients who underwent robotic surgery for rectal cancer, there was no difference among the easy, moderate, and difficult groups in terms of operative time, incidences of postoperative complications or pathological outcomes. The study concluded that robotic surgery could provide increased comfort for surgeons even with difficult pelvic anatomy, and might overcome the difficutly [31]. Therefore, robotic surgery might also be a more suitable approach for radical surgery to treat rectal GIST than open or laparoscopic surgery.

According to the Japanese Clinical Practice Guidelines for GISTs [13], there is no clear basis or consensus to recommend the preoperative use of neoadjuvant imatinib therapy for resectable GIST. However, this intervention may be considered for advanced rectal GIST to preserve organ function. A recent retrospective multi-institutional study reported that neoadjuvant imatinib therapy could shrink rectal GIST, increasing the rate of R0 resection and sphincter preservation, with no significant differences in RFS between patients who received or did not receive neoadjuvant imatinib therapy [32]. In this study, only two patients underwent neoadjuvant imatinib therapy. One task is to identify which cases are suitable for neoadjuvant imatinib therapy. Smaller tumors are more likely to achieve R0 and to preserve sphincter. Aggressive neoadjuvant imatinib therapy might be acceptable for relatively large tumors.

Transanal total mesorectal excision (taTME) was not performed for rectal tumors at our institution. A study with 21 patients reported that taTME is useful for rectal GIST [33]. Furthermore, two-team surgery might be superior to one-team surgery in terms of operative time and blood loss. Therefore, a two-team transanal approach might also be an effective option for rectal GIST.

This study has several limitations. First, this was a retrospective study conducted at a single institution with a small number of patients. Second, patients undergoing laparoscopic or open surgery were not enrolled. Third, postoperative anal function was not objectively evaluated. However, none of the patients underwent recreation of a stoma due to dyschezia. Fourth, although median follow-up time was 76 months, 2 patients had less than 12 months of follow up while the other 11 patients had follow-up that was longer than 24 months.

In conclusion, we demonstrated favorable short-term and long-term outcomes after radical robotic surgery for rectal GIST. Rectal GIST is extremely rare; therefore, a meta-analysis is needed to confirm the optimal approach for radical resection of rectal GIST.

### Supplementary Information

Supplementary Material 1.

## Data Availability

The datasets used and analyzed during the current study are available from the corresponding author on reasonable request.
