# Supplementary material for: Short-term and long-term outcomes after robotic radical surgery for rectal gastrointestinal stromal tumor
Source: BMC Surg. 2024 May 9;24:141. doi: 10.1186/s12893-024-02434-y (PMC11080177; doi:10.1186/s12893-024-02434-y)
Supplement: Supplementary file 1 — Supplementary Material 1. [file 12893_2024_2434_MOESM1_ESM.zip › Supplemental video.docx]

**Supplemental video of robotic radical surgery for rectal GIST.**

The case is a 65-year-old man. We performed robotic intersphincteric resection for rectal GIST. Mobilization in the medial to lateral approach gets started at the iliac arterial bifurcation. When the mesorectum becomes mobilized, it is pulled up by gauze looped around it for mobilizing the rectum. After the posterior rectal wall in mobilized, the left and right rectal walls become mobilized, too. The anterior rectal wall also gets mobilized, and dissection proceeds very carefully toward the anus. Sharp dissection is done to avoid autonomic nerve injury while mobilizing the rectum. After getting the posterior rectal wall mobilized further, resection of the anococcygeal ligament is done. The dissection proceeds further beyond the tumor on the anus side, robotic approach is completed, and perineal manipulation is started.
